# Crystal structure and Hirshfeld analysis of di-*tert*-butyl 2,2′-[(ethyl­aza­nedi­yl)bis­(methyl­ene)]bis­(1*H*-pyrrole-1-carboxyl­ate)

**DOI:** 10.1107/S2056989020014966

**Published:** 2020-11-13

**Authors:** Elizaveta A. Kvyatkovskaya, Zeliha Atioğlu, Mehmet Akkurt, Polina P. Epifanova, Karina S. Valchuk, Victor N. Khrustalev, Ajaya Bhattarai

**Affiliations:** a Faculty of Science, Peoples’ Friendship University of Russia (RUDN University), 6 Miklukho-Maklaya St, Moscow, 117198, Russian Federation; b İlke Education and Health Foundation, Cappadocia University, Cappadocia Vocational College, The Medical Imaging Techniques Program, 50420 Mustafapaşa, Ürgüp, Nevşehir, Turkey; c Department of Physics, Faculty of Sciences, Erciyes University, 38039 Kayseri, Turkey; d N. D. Zelinsky Institute of Organic Chemistry RAS, Leninsky Prosp. 47, Moscow, 119991 , Russian Federation; e Department of Chemistry, M.M.A.M.C (Tribhuvan University), Biratnagar, Nepal

**Keywords:** crystal structure, pyrrole ring, C—H⋯π inter­actions, π–π stacking inter­actions, Hirshfeld surface analysis

## Abstract

The title compound, C_22_H_33_N_3_O_4_, crystallizes in the triclinic space group *P*  with two mol­ecules in a unit cell. The two pyrrole rings are essentially planar (r.m.s. deviation = 0.002 Å) and they form a dihedral angle of 81.24 (10)° with each other. The crystal packing is stabilized by C—H⋯π inter­actions and π–π stacking inter­actions, forming a three-dimensional network. The Hirshfeld surface analysis and two-dimensional fingerprint plots reveal that the most important contributions for the crystal packing are from H⋯H (74.3%), C⋯H/H⋯C (11.5%) and O⋯H/H⋯O (9.1%) contacts.

## Abstract 2

In the title compound, inter­molecular C—H⋯π inter­actions and π–π stacking inter­actions help to stabilize the crystal structure, forming a three-dimensional network.

## Chemical context   

**Figure 1:**
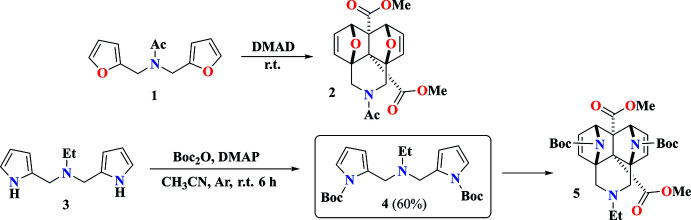
Reaction scheme including the title compound **4** as inter­mediate.

This work is a continuation of the study of Diels–Alder reactions on bis-diene systems, which was previously carried out on the example of the tandem [4 + 2]/[4 + 2] cyclo­addition between bis-furyl dienes similar to **1** and activated alkynes, leading to adducts such as **2**, as shown in Fig. 1 ▸ (Borisova *et al.*, 2018*a* ▸,*b* ▸; Kvyatkovskaya *et al.*, 2020 ▸; Lautens & Fillion, 1997 ▸; Domingo *et al.*, 2000 ▸). Here we aimed to investigate substrates containing two pyrrole moieties under the same reaction conditions. For this reason, *N*,*N*-*bis*(1*H*-pyrrol-2-ylmeth­yl) ethanamine (**3**) was synthesized using a Mannich reaction according to the described procedure (Raines & Kovacs, 1970 ▸). It is known that pyrrole fragments are capable of reacting with the most active dienophiles in the [4 + 2] cyclo­addition reaction, which requires the presence of electron-deficient groups at the nitro­gen atom (Winkler, 1996 ▸; Visnick & Battiste, 1985 ▸; Butler *et al.*, 2000 ▸; Warrener *et al.*, 2003 ▸). Thus, the pyrrole rings of amine **3** were activated by Boc-protecting groups to give the title substance **4**. Considering that a single example of a successful domino [4 + 2] cyclo­addition between hexa­fluoro­but-2-yne and *N*,*N*′-dipyrrolyl­methane is reported in the literature (Visnick & Battiste, 1985 ▸), we tested amine **4** in the reaction with such an active dienophile as dimethyl acetyl­enedi­carboxyl­ate (DMAD). The experiments were performed in a wide temperature range (from room temperature to 413 K) and led to multicomponent mixtures of products at elevated temperatures, from which we were unable to isolate the target adduct **5**.
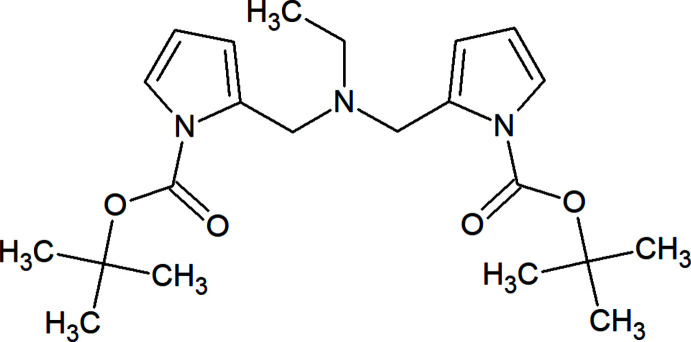


However, taking into account the importance of the non-covalent bond-donor/acceptor properties of the nitro­gen atom in N-heterocycles for synthesis, catalysis and the design of new materials (Asadov *et al.*, 2016 ▸; Gurbanov *et al.*, 2017 ▸, 2018*a* ▸,*b* ▸; Karmakar *et al.*, 2017 ▸; Maharramov *et al.*, 2018 ▸; Mahmoudi *et al.*, 2017 ▸, 2019 ▸; Mahmudov *et al.*, 2010 ▸, 2013 ▸, 2017*a* ▸,*b* ▸, 2019 ▸, 2020 ▸; Shixaliyev *et al.*, 2014 ▸), we describe in this work the structural features of compound **4**.

## Structural commentary   

**Figure 2:**
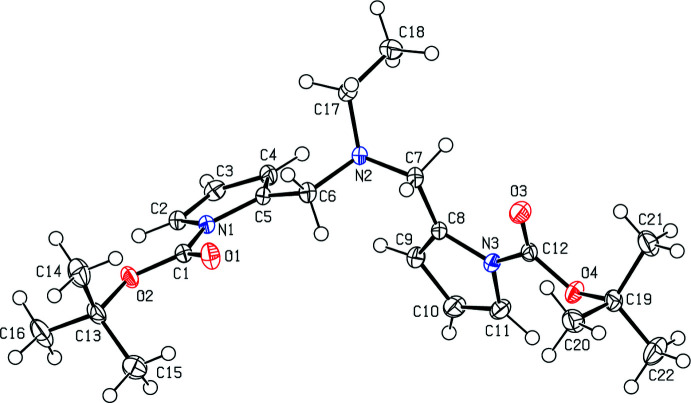
The mol­ecular structure of the title compound **4** with displacement ellipsoids for the non-hydrogen atoms drawn at the 50% probability level.

As shown in Fig. 2 ▸, the two pyrrole rings (N1/C2–C5 and N3/C8–C11) in the title compound **4** form a dihedral angle of 81.24 (10)°. The C6—N2—C17—C18 and C7—N2—C17—C18, C5—C6—N2—C17, C8—C7—N2—C17 and C6—N2—C7—C8 torsion angles are −163.52 (15), 71.9 (2), −87.35 (17), −155.20 (14) and 80.67 (16)°, respectively. All of the bond lengths and angles in the title compound **4** are of usual values.

## Supra­molecular features   

**Figure 3:**
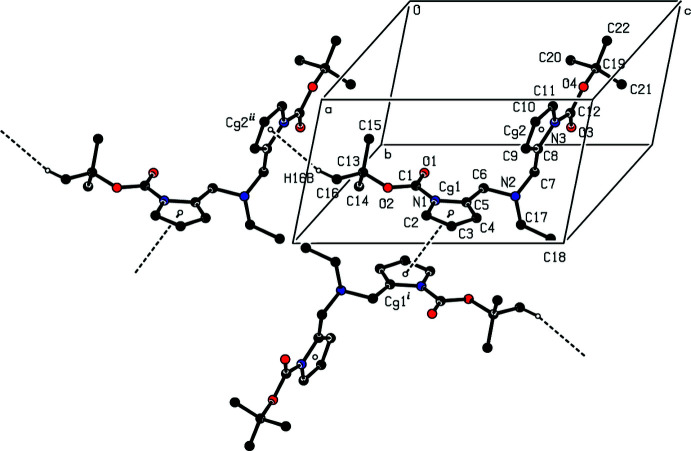
A view of the inter­molecular C—H⋯π inter­actions and π–π- stacking inter­actions of the title compound **4**. Symmetry codes: (i) 2 − *x*, 2 − *y*, 1 − *z*; (ii) *x*, *y*, −1 + *z*.

The supra­molecular structure of the title compound **4** is defined by π–π stacking [*Cg*1⋯*Cg*1^i^ = 3.6892 (13) Å, symmetry code (i): 2 − *x*, 2 − *y*, 1 − *z*, slippage = 1.794 Å, where *Cg*1 is the centroid of the N1/C2–C5 pyrrole ring] and C—H⋯π [C16—H16*B*⋯*Cg*2^ii^, symmetry code (ii): *x*, *y*, −1 + *z*, where *Cg*2 is the centroid of the N3/C8–C11 pyrrole ring] inter­actions, forming a three-dimensional network (Fig. 3 ▸; Table 1 ▸). There are no conventional hydrogen bonds in the structure.

## Hirshfeld surface analysis   

**Figure 4:**
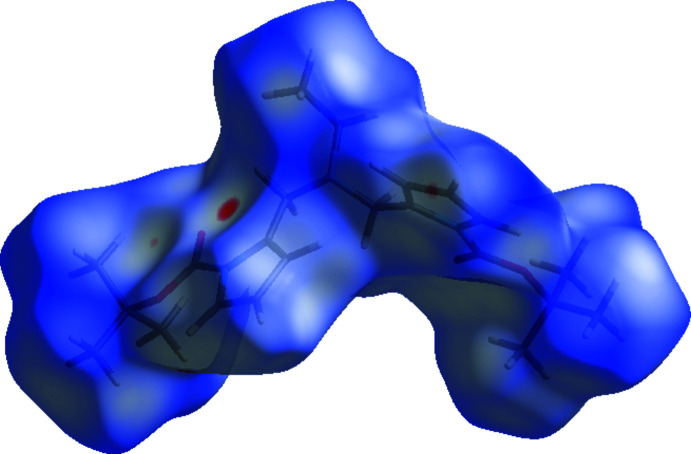
A view of the three-dimensional Hirshfeld surface for the title compound **4**, plotted over *d*_norm_ in the range −0.0919 to 1.6027 a.u.

The Hirshfeld surface analysis (Spackman & Jayatilaka, 2009 ▸) was performed and the associated two-dimensional fingerprint plots (McKinnon, *et al.*, 2007 ▸) were obtained with *Crystal Explorer17* (Turner *et al.*, 2017 ▸) to investigate the inter­molecular inter­actions and surface morphology. The Hirshfeld surface mapped over *d*_norm_ using a standard surface resolution with a fixed colour scale of −0.0919 (red) to 1.6027 (blue) a.u. is shown in Fig. 4 ▸.

**Figure 5:**
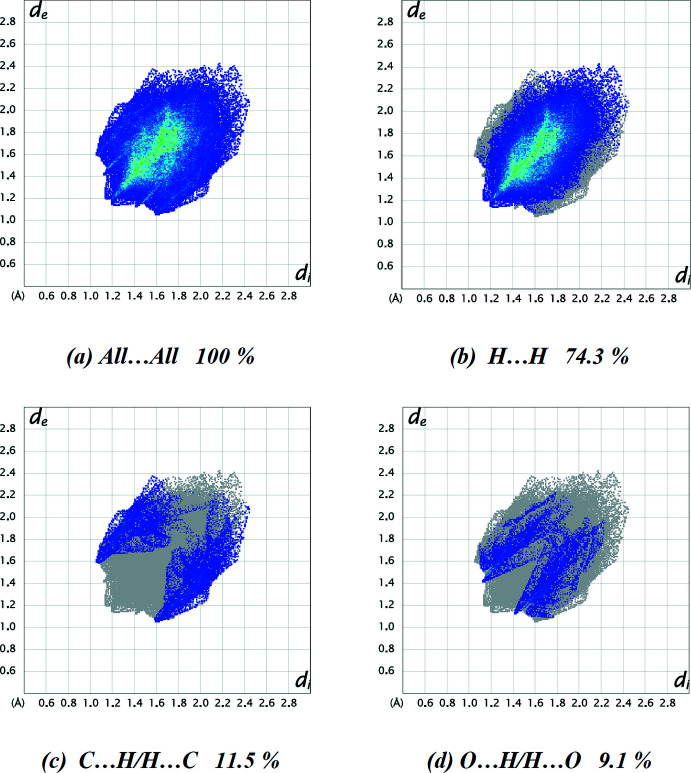
A view of the two-dimensional fingerprint plots for the title compound **4**, showing (*a*) all inter­actions, and delineated into (*b*) H⋯H, (*c*) C⋯H/H⋯C and (*d*) O⋯H/H⋯O inter­actions. The *d*_i_ and *d*_e_ values are the closest inter­nal and external distances (in Å) from given points on the Hirshfeld surface contacts.

The percentage contributions of various contacts (Table 2 ▸) to the total Hirshfeld surface are listed in Table 3 ▸ and shown in the two-dimensional fingerprint plots in Fig. 5 ▸, revealing that the crystal packing is dominated by H⋯H contacts, representing van der Waals inter­actions (74.3% contribution to the overall surface), followed by C⋯H/H⋯C and O⋯H/H⋯O inter­actions, which contribute 11.5% and 9.1%, respectively.

## Database survey   

A search of the Cambridge Structural Database (CSD, Version 5.39, update of August 2018; Groom *et al.*, 2016 ▸) using *Conquest* (Bruno *et al.*, 2002 ▸) for the *di-tert-*butyl 2,2′-[(ethyl­aza­nedi­yl)bis­(methyl­ene)[bis­(1*H*-pyrrole-1-carboxyl­ate)] skeleton revealed 37 structures similar to the title compound **4**. Only three of them are closely related to the title compound, *viz*. di-*tert*-butyl 2,2′-(anthracene-9,10-di­yl)bis(1*H*-pyrrole-1-carboxyl­ate) in the space group *P*2_1_/*n* (CSD refcode PUKKEO; Wang *et al.*, 2020 ▸), *tert*-butyl 2-{4-[1-(*tert*-but­oxy­carbon­yl)-1*H*-pyrrol-2-yl]-2,5-bis (2,2-di­cyano­vin­yl)phen­yl}-1*H*-pyrrole-1-carboxyl­ate in the space group *C*2/c (IVIJAA; Zhang *et al.*, 2017 ▸) and bis­(3-bromo-1- (*tert*-butyl­oxycarbon­yl)-5-(meth­oxy­carbon­yl)-pyrrol-2-yl)methane in the space group *P* 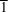 (NANLAP; Kitamura & Yamashita, 1997 ▸).

In the crystal of PUKKEO, the distance between two parallel mol­ecules within one column was measured to be 9.333 Å, indicating that π–π inter­actions cannot be formed in the mol­ecule. In the crystal structure of IVIJAA, multiple inter­molecular C—H⋯N (or C—H⋯O) and C—H⋯π inter­actions were found, which could help to rigidify the mol­ecular conformation. In NANLAP, the dihedral angle between the two pyrrole ring is 82.77°.

In the three structures closely related to the title compound, the different linkers between the two pyrrole units (aromatic *vs* aliphatic, large *vs* small) may account for the distinct inter­molecular inter­actions in the crystals.

## Synthesis and crystallization   

*Di*-*tert*-butyl dicarbonate [(Boc)_2_O, 27.8 mL, 0.13 mol] was added to a solution of *N*,*N*-*bis*(1*H*-pyrrol-2-ylmeth­yl)ethanamine (12.0 g, 0.06 mol) and DMAP (1.1 g, 0.009 mol) in CH_3_CN (50 mL) at room temperature under an argon atmosphere. The mixture was stirred for 6 h at room temperature. The reaction mixture was poured into a 5% solution of NH_3_ in H_2_O (300 mL) and extracted with CH_2_Cl_2_ (3 × 50 mL). The combined organic layers were dried over MgSO_4_, filtered and concentrated. Flash chromatography purification on alumin­ium oxide (hexa­ne) of the residue yielded the title compound as colourless crystals. Single crystals suitable for X-ray diffraction analysis were obtained by slow evaporation of an EtOAc/hexane solution at room temperature. Colourless prisms. Yield 14.25 g (60%). M.p. = 349.8–351.5 K (hexane, Al_2_O_3_). IR (KBr), ν (cm^−1^): 3112, 3172. ^1^H NMR (CDCl_3_, 600.1 MHz): δ = 1.08 (*t*, 3H, NCH_2_CH_3_, *J* = 6.6), 1.57 (*s*, 18H, 2 × ^*t*^Bu), 2.67 (*q*, 2H, N–CH_2_–CH_3_, *J* = 6.6), 3.90 (*s*, 4H, 2 × N–CH_2_), 6.09 (*t*, 2H, H-4, pyrrole, *J* = 3.3), 6.31 (*m*, 2H, H-3, pyrrole), 7.16 (*dd*, 2H, H-5, pyrrole, *J* = 1.7, J = 3.3). ^13^C NMR (100.6 MHz, CDCl_3_): δ = 12.6 (NCH_2_CH_3_), 28.1 [2C, 2 × C(CH_3_)_3_], 48.9 (N–CH_2_–CH_3_), 52.8 (2C, CH_2_–N–CH_2_), 83.3 [2C, 2 × O–C(CH_3_)_3_], 110.2 (2C, 2 × C-3, pyrrole), 111.7 (2C, 2 × C-4, pyrrole), 120.9 (2C, 2 × C-5, pyrrole), 134.9 (2C, 2 × C-2, pyrrole), 149.5 (2C, 2 × CO). Elemental analysis calculated for C_22_H_33_N_3_O_4_ (%): C 65.12, H 7.88, N 10.73; found (%): C 65.48, H 8.24, N 10.41.

## Refinement   

Crystal data, data collection and structure refinement details are summarized in Table 4 ▸. All H atoms were included as riding contributions in idealized positions (C—H = 0.95–0.99 Å with *U*_iso_(H) = 1.2 or 1.5*U*_eq_(C).

## Supplementary Material

Crystal structure: contains datablock(s) I. DOI: 10.1107/S2056989020014966/yz2002sup1.cif

Structure factors: contains datablock(s) I. DOI: 10.1107/S2056989020014966/yz2002Isup2.hkl

Supporting information file. DOI: 10.1107/S2056989020014966/yz2002Isup3.cml

CCDC reference: 2043609

Additional supporting information:  crystallographic information; 3D view; checkCIF report

## Figures and Tables

**Table 1:** Hydrogen-bond geometry (Å, °) *Cg*2 is the centroid of the N3/C8–C11 pyrrole ring.

*D*—H⋯*A*	*D*—H	H⋯*A*	*D*⋯*A*	*D*—H⋯*A*
C16—H16*B*⋯*Cg*2^i^	0.98	2.85	3.779 (2)	158

Symmetry code: (i) 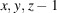.

**Table 2:** Summary of short inter­atomic contacts (Å) in the title compound **4**

Contact	Distance	Symmetry operation
H17*A*⋯O1	2.73	1 − *x*, 2 − *y*, 1 − *z*
H22*B*⋯O1	2.72	−*x*, 1 − *y*, 1 − *z*
H22*A*⋯H2	2.59	1 − *x*, 1 − *y*, 1 − *z*
H20*B*⋯H10	2.48	−1 + *x*, *y*, *z*
C8⋯H16*B*	2.75	*x*, *y*, 1 + *z*
H16*A*⋯C18	3.06	2 − *x*, 2 − *y*, 1 − *z*
H18*C*⋯C21	2.96	1 − *x*, 2 − *y*, 2 − *z*
H18*A*⋯H18*A*	2.58	2 − *x*, 2 − *y*, 2 − *z*

**Table 3:** Percentage contributions of inter­atomic contacts to the Hirshfeld surface for the title compound **4**

Contact	Percentage contribution
H⋯H	74.3
C⋯H/H⋯C	11.5
O⋯H/H⋯O	9.1
N⋯H/H⋯N	3.4
N⋯C/C⋯N	0.7
O⋯C/C⋯O	0.5
C⋯C	0.5

**Table 4:** Experimental details

Crystal data	
Chemical formula	C_22_H_33_N_3_O_4_
*M*_r_	403.51
Crystal system, space group	Triclinic, *P* 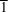
Temperature (K)	100
*a*, *b*, *c* (Å)	9.6579 (19), 11.798 (2), 12.216 (2)
α, β, γ (°)	100.95 (3), 109.41 (3), 111.12 (3)
*V* (Å^3^)	1146.3 (7)
*Z*	2
Radiation type	Synchrotron, λ = 0.96990 Å
μ (mm^−1^)	0.17
Crystal size (mm)	0.25 × 0.15 × 0.12

Data collection	
Diffractometer	Rayonix SX165 CCD
Absorption correction	Multi-scan (*SCALA*; Evans, 2006 ▸)
*T*_min_, *T*_max_	0.950, 0.970
No. of measured, independent and observed [*I* > 2σ(*I*)] reflections	14236, 4609, 3323
*R*_int_	0.081
(sin θ/λ)_max_ (Å^−1^)	0.642

Refinement	
*R*[*F* ^2^ > 2σ(*F* ^2^)], *wR*(*F* ^2^), *S*	0.067, 0.180, 1.04
No. of reflections	4609
No. of parameters	270
H-atom treatment	H-atom parameters constrained
Δρ_max_, Δρ_min_ (e Å^−3^)	0.36, −0.32

Computer programs: *Marccd* (Doyle, 2011 ▸), *iMosflm* (Battye *et al.*, 2011 ▸), *SHELXT* (Sheldrick, 2015*a* ▸), *SHELXL* (Sheldrick, 2015*b* ▸), *ORTEP-3 for Windows* (Farrugia, 2012 ▸) and *PLATON* (Spek, 2020 ▸).

## supplementary crystallographic information

### Crystal data

**Table:**

C_22_H_33_N_3_O_4_	*Z* = 2
*M**_r_* = 403.51	*F*(000) = 436
Triclinic, *P*1	*D*_x_ = 1.169 Mg m^−^^3^
*a* = 9.6579 (19) Å	Synchrotron radiation, λ = 0.96990 Å
*b* = 11.798 (2) Å	Cell parameters from 600 reflections
*c* = 12.216 (2) Å	θ = 3.4–34.0°
α = 100.95 (3)°	µ = 0.17 mm^−^^1^
β = 109.41 (3)°	*T* = 100 K
γ = 111.12 (3)°	Prism, colourless
*V* = 1146.3 (7) Å^3^	0.25 × 0.15 × 0.12 mm

### Data collection

**Table:**

Rayonix SX165 CCD diffractometer	3323 reflections with *I* > 2σ(*I*)
/f scan	*R*_int_ = 0.081
Absorption correction: multi-scan (Scala; Evans, 2006)	θ_max_ = 38.5°, θ_min_ = 3.4°
*T*_min_ = 0.950, *T*_max_ = 0.970	*h* = −11→11
14236 measured reflections	*k* = −15→15
4609 independent reflections	*l* = −15→10

### Refinement

**Table:**

Refinement on *F*^2^	Secondary atom site location: difference Fourier map
Least-squares matrix: full	Hydrogen site location: inferred from neighbouring sites
*R*[*F*^2^ > 2σ(*F*^2^)] = 0.067	H-atom parameters constrained
*wR*(*F*^2^) = 0.180	*w* = 1/[σ^2^(*F*_o_^2^) + (0.0405*P*)^2^] where *P* = (*F*_o_^2^ + 2*F*_c_^2^)/3
*S* = 1.04	(Δ/σ)_max_ < 0.001
4609 reflections	Δρ_max_ = 0.36 e Å^−^^3^
270 parameters	Δρ_min_ = −0.32 e Å^−^^3^
0 restraints	Extinction correction: SHELXL, Fc^*^=kFc[1+0.001xFc^2^λ^3^/sin(2θ)]^-1/4^
Primary atom site location: difference Fourier map	Extinction coefficient: 0.039 (3)

### Special details

**Table:**

Geometry. All esds (except the esd in the dihedral angle between two l.s. planes) are estimated using the full covariance matrix. The cell esds are taken into account individually in the estimation of esds in distances, angles and torsion angles; correlations between esds in cell parameters are only used when they are defined by crystal symmetry. An approximate (isotropic) treatment of cell esds is used for estimating esds involving l.s. planes.

### Fractional atomic coordinates and isotropic or equivalent isotropic displacement parameters (Å^2^)

**Table:**

	*x*	*y*	*z*	*U*_iso_*/*U*_eq_	
O1	0.54208 (15)	0.82396 (12)	0.31750 (10)	0.0262 (4)	
O2	0.71965 (15)	0.80484 (11)	0.23867 (10)	0.0227 (3)	
O3	0.22197 (15)	0.72623 (11)	0.74478 (11)	0.0245 (3)	
O4	0.11894 (14)	0.51479 (10)	0.73408 (11)	0.0222 (3)	
N1	0.79450 (17)	0.84298 (12)	0.44180 (12)	0.0172 (4)	
N2	0.67247 (17)	0.89738 (12)	0.70376 (12)	0.0194 (4)	
N3	0.35254 (17)	0.60263 (12)	0.71412 (12)	0.0179 (4)	
C1	0.6716 (2)	0.82405 (15)	0.32830 (15)	0.0196 (4)	
C2	0.9456 (2)	0.84337 (15)	0.45916 (15)	0.0194 (4)	
H2	0.9835	0.8329	0.3972	0.023*	
C3	1.0283 (2)	0.86130 (15)	0.58039 (16)	0.0221 (4)	
H3	1.1340	0.8651	0.6182	0.027*	
C4	0.9274 (2)	0.87346 (15)	0.64092 (15)	0.0212 (4)	
H4	0.9553	0.8871	0.7262	0.025*	
C5	0.7845 (2)	0.86223 (14)	0.55571 (15)	0.0187 (4)	
C6	0.6378 (2)	0.86467 (16)	0.57186 (15)	0.0218 (4)	
H6A	0.6124	0.9298	0.5408	0.026*	
H6B	0.5404	0.7786	0.5230	0.026*	
C7	0.5214 (2)	0.83163 (15)	0.71812 (15)	0.0198 (4)	
H7A	0.4270	0.8326	0.6541	0.024*	
H7B	0.5353	0.8777	0.8007	0.024*	
C8	0.4879 (2)	0.69338 (15)	0.70485 (14)	0.0177 (4)	
C9	0.5758 (2)	0.63131 (16)	0.68252 (15)	0.0218 (4)	
H9	0.6736	0.6684	0.6721	0.026*	
C10	0.4954 (2)	0.50002 (16)	0.67744 (16)	0.0253 (5)	
H10	0.5308	0.4355	0.6633	0.030*	
C11	0.3603 (2)	0.48460 (15)	0.69642 (16)	0.0230 (5)	
H11	0.2841	0.4072	0.6975	0.028*	
C12	0.2268 (2)	0.62378 (15)	0.73226 (14)	0.0177 (4)	
C13	0.6074 (2)	0.77552 (16)	0.10726 (15)	0.0235 (5)	
C14	0.5794 (3)	0.89213 (19)	0.09584 (17)	0.0336 (5)	
H14A	0.5206	0.9074	0.1442	0.050*	
H14B	0.5135	0.8755	0.0085	0.050*	
H14C	0.6861	0.9688	0.1274	0.050*	
C15	0.4477 (2)	0.65187 (18)	0.06423 (17)	0.0341 (5)	
H15A	0.4740	0.5843	0.0870	0.051*	
H15B	0.3852	0.6221	−0.0260	0.051*	
H15C	0.3812	0.6699	0.1041	0.051*	
C16	0.7089 (3)	0.7536 (2)	0.04130 (17)	0.0363 (6)	
H16A	0.8146	0.8318	0.0752	0.055*	
H16B	0.6486	0.7344	−0.0475	0.055*	
H16C	0.7292	0.6803	0.0536	0.055*	
C17	0.7538 (2)	1.03940 (15)	0.76618 (16)	0.0247 (5)	
H17A	0.6706	1.0716	0.7420	0.030*	
H17B	0.8378	1.0801	0.7377	0.030*	
C18	0.8364 (2)	1.08057 (17)	0.90690 (16)	0.0323 (5)	
H18A	0.9061	1.0379	0.9308	0.048*	
H18B	0.7517	1.0554	0.9371	0.048*	
H18C	0.9047	1.1750	0.9433	0.048*	
C19	−0.0459 (2)	0.49847 (16)	0.72438 (15)	0.0208 (4)	
C20	−0.1389 (2)	0.50783 (18)	0.60138 (16)	0.0284 (5)	
H20A	−0.0858	0.5970	0.6037	0.043*	
H20B	−0.2534	0.4839	0.5870	0.043*	
H20C	−0.1373	0.4486	0.5342	0.043*	
C21	−0.0258 (2)	0.59732 (18)	0.83598 (17)	0.0304 (5)	
H21A	0.0432	0.5918	0.9123	0.046*	
H21B	−0.1345	0.5791	0.8332	0.046*	
H21C	0.0267	0.6848	0.8343	0.046*	
C22	−0.1245 (2)	0.36136 (17)	0.72450 (19)	0.0353 (5)	
H22A	−0.1313	0.3003	0.6539	0.053*	
H22B	−0.2357	0.3392	0.7179	0.053*	
H22C	−0.0572	0.3562	0.8018	0.053*	

### Atomic displacement parameters (Å^2^)

**Table:**

	*U*^11^	*U*^22^	*U*^33^	*U*^12^	*U*^13^	*U*^23^
O1	0.0223 (8)	0.0387 (8)	0.0215 (7)	0.0145 (6)	0.0118 (6)	0.0133 (6)
O2	0.0238 (7)	0.0281 (7)	0.0129 (6)	0.0086 (5)	0.0090 (5)	0.0056 (5)
O3	0.0240 (7)	0.0220 (6)	0.0296 (7)	0.0103 (5)	0.0139 (6)	0.0099 (5)
O4	0.0179 (7)	0.0214 (6)	0.0287 (7)	0.0063 (5)	0.0134 (6)	0.0110 (5)
N1	0.0181 (8)	0.0174 (7)	0.0145 (8)	0.0058 (6)	0.0084 (6)	0.0053 (6)
N2	0.0220 (8)	0.0167 (7)	0.0179 (8)	0.0044 (6)	0.0127 (6)	0.0049 (6)
N3	0.0179 (8)	0.0159 (7)	0.0203 (8)	0.0055 (6)	0.0113 (6)	0.0063 (6)
C1	0.0212 (10)	0.0187 (8)	0.0155 (9)	0.0048 (7)	0.0093 (8)	0.0049 (7)
C2	0.0197 (10)	0.0190 (8)	0.0192 (9)	0.0066 (7)	0.0111 (8)	0.0056 (7)
C3	0.0197 (10)	0.0198 (8)	0.0243 (10)	0.0075 (7)	0.0084 (8)	0.0080 (7)
C4	0.0221 (10)	0.0230 (9)	0.0146 (9)	0.0064 (7)	0.0080 (8)	0.0070 (7)
C5	0.0234 (10)	0.0152 (8)	0.0172 (9)	0.0052 (7)	0.0126 (8)	0.0058 (7)
C6	0.0251 (10)	0.0213 (9)	0.0183 (9)	0.0075 (8)	0.0115 (8)	0.0080 (7)
C7	0.0210 (10)	0.0212 (9)	0.0183 (9)	0.0076 (7)	0.0115 (8)	0.0081 (7)
C8	0.0181 (9)	0.0172 (8)	0.0142 (8)	0.0036 (7)	0.0083 (7)	0.0048 (7)
C9	0.0204 (10)	0.0219 (9)	0.0240 (10)	0.0075 (7)	0.0132 (8)	0.0083 (7)
C10	0.0256 (11)	0.0209 (9)	0.0336 (11)	0.0119 (8)	0.0167 (9)	0.0085 (8)
C11	0.0227 (10)	0.0167 (8)	0.0283 (10)	0.0064 (7)	0.0124 (8)	0.0084 (7)
C12	0.0169 (10)	0.0192 (8)	0.0130 (9)	0.0045 (7)	0.0063 (7)	0.0056 (7)
C13	0.0251 (11)	0.0303 (10)	0.0111 (9)	0.0103 (8)	0.0070 (8)	0.0054 (7)
C14	0.0429 (13)	0.0428 (11)	0.0246 (10)	0.0239 (10)	0.0172 (9)	0.0183 (9)
C15	0.0314 (12)	0.0355 (11)	0.0197 (10)	0.0059 (9)	0.0059 (9)	0.0058 (8)
C16	0.0389 (13)	0.0514 (13)	0.0177 (10)	0.0198 (10)	0.0150 (9)	0.0072 (9)
C17	0.0285 (11)	0.0173 (8)	0.0279 (10)	0.0060 (8)	0.0174 (9)	0.0070 (8)
C18	0.0308 (12)	0.0261 (10)	0.0265 (11)	0.0027 (8)	0.0141 (9)	−0.0002 (8)
C19	0.0157 (10)	0.0258 (9)	0.0222 (9)	0.0084 (7)	0.0115 (8)	0.0072 (7)
C20	0.0225 (11)	0.0332 (10)	0.0230 (10)	0.0087 (8)	0.0094 (8)	0.0057 (8)
C21	0.0290 (11)	0.0392 (11)	0.0231 (10)	0.0138 (9)	0.0153 (9)	0.0076 (9)
C22	0.0283 (12)	0.0315 (10)	0.0507 (13)	0.0098 (9)	0.0239 (10)	0.0193 (10)

### Geometric parameters (Å, º)

**Table:**

O1—C1	1.213 (2)	C11—H11	0.9500
O2—C1	1.336 (2)	C13—C14	1.517 (3)
O2—C13	1.495 (2)	C13—C16	1.519 (3)
O3—C12	1.209 (2)	C13—C15	1.527 (3)
O4—C12	1.3415 (19)	C14—H14A	0.9800
O4—C19	1.494 (2)	C14—H14B	0.9800
N1—C2	1.401 (2)	C14—H14C	0.9800
N1—C1	1.407 (2)	C15—H15A	0.9800
N1—C5	1.408 (2)	C15—H15B	0.9800
N2—C7	1.472 (2)	C15—H15C	0.9800
N2—C6	1.473 (2)	C16—H16A	0.9800
N2—C17	1.475 (2)	C16—H16B	0.9800
N3—C12	1.401 (2)	C16—H16C	0.9800
N3—C11	1.401 (2)	C17—C18	1.523 (3)
N3—C8	1.415 (2)	C17—H17A	0.9900
C2—C3	1.360 (2)	C17—H17B	0.9900
C2—H2	0.9500	C18—H18A	0.9800
C3—C4	1.434 (3)	C18—H18B	0.9800
C3—H3	0.9500	C18—H18C	0.9800
C4—C5	1.366 (2)	C19—C22	1.520 (2)
C4—H4	0.9500	C19—C21	1.521 (3)
C5—C6	1.503 (2)	C19—C20	1.521 (2)
C6—H6A	0.9900	C20—H20A	0.9800
C6—H6B	0.9900	C20—H20B	0.9800
C7—C8	1.508 (2)	C20—H20C	0.9800
C7—H7A	0.9900	C21—H21A	0.9800
C7—H7B	0.9900	C21—H21B	0.9800
C8—C9	1.364 (3)	C21—H21C	0.9800
C9—C10	1.439 (2)	C22—H22A	0.9800
C9—H9	0.9500	C22—H22B	0.9800
C10—C11	1.354 (3)	C22—H22C	0.9800
C10—H10	0.9500		
			
C1—O2—C13	120.54 (15)	C14—C13—C15	113.23 (17)
C12—O4—C19	121.12 (14)	C16—C13—C15	111.00 (16)
C2—N1—C1	125.39 (14)	C13—C14—H14A	109.5
C2—N1—C5	109.01 (14)	C13—C14—H14B	109.5
C1—N1—C5	125.59 (15)	H14A—C14—H14B	109.5
C7—N2—C6	111.01 (13)	C13—C14—H14C	109.5
C7—N2—C17	112.51 (14)	H14A—C14—H14C	109.5
C6—N2—C17	110.28 (13)	H14B—C14—H14C	109.5
C12—N3—C11	125.04 (14)	C13—C15—H15A	109.5
C12—N3—C8	126.37 (14)	C13—C15—H15B	109.5
C11—N3—C8	108.54 (14)	H15A—C15—H15B	109.5
O1—C1—O2	127.13 (16)	C13—C15—H15C	109.5
O1—C1—N1	123.13 (15)	H15A—C15—H15C	109.5
O2—C1—N1	109.73 (16)	H15B—C15—H15C	109.5
C3—C2—N1	107.86 (15)	C13—C16—H16A	109.5
C3—C2—H2	126.1	C13—C16—H16B	109.5
N1—C2—H2	126.1	H16A—C16—H16B	109.5
C2—C3—C4	107.70 (17)	C13—C16—H16C	109.5
C2—C3—H3	126.2	H16A—C16—H16C	109.5
C4—C3—H3	126.2	H16B—C16—H16C	109.5
C5—C4—C3	108.77 (15)	N2—C17—C18	112.62 (15)
C5—C4—H4	125.6	N2—C17—H17A	109.1
C3—C4—H4	125.6	C18—C17—H17A	109.1
C4—C5—N1	106.67 (16)	N2—C17—H17B	109.1
C4—C5—C6	129.41 (15)	C18—C17—H17B	109.1
N1—C5—C6	123.90 (15)	H17A—C17—H17B	107.8
N2—C6—C5	110.12 (14)	C17—C18—H18A	109.5
N2—C6—H6A	109.6	C17—C18—H18B	109.5
C5—C6—H6A	109.6	H18A—C18—H18B	109.5
N2—C6—H6B	109.6	C17—C18—H18C	109.5
C5—C6—H6B	109.6	H18A—C18—H18C	109.5
H6A—C6—H6B	108.2	H18B—C18—H18C	109.5
N2—C7—C8	109.26 (14)	O4—C19—C22	102.03 (14)
N2—C7—H7A	109.8	O4—C19—C21	110.41 (14)
C8—C7—H7A	109.8	C22—C19—C21	111.25 (15)
N2—C7—H7B	109.8	O4—C19—C20	108.05 (14)
C8—C7—H7B	109.8	C22—C19—C20	111.12 (15)
H7A—C7—H7B	108.3	C21—C19—C20	113.35 (16)
C9—C8—N3	107.00 (14)	C19—C20—H20A	109.5
C9—C8—C7	128.71 (15)	C19—C20—H20B	109.5
N3—C8—C7	124.28 (16)	H20A—C20—H20B	109.5
C8—C9—C10	108.38 (16)	C19—C20—H20C	109.5
C8—C9—H9	125.8	H20A—C20—H20C	109.5
C10—C9—H9	125.8	H20B—C20—H20C	109.5
C11—C10—C9	107.95 (16)	C19—C21—H21A	109.5
C11—C10—H10	126.0	C19—C21—H21B	109.5
C9—C10—H10	126.0	H21A—C21—H21B	109.5
C10—C11—N3	108.12 (15)	C19—C21—H21C	109.5
C10—C11—H11	125.9	H21A—C21—H21C	109.5
N3—C11—H11	125.9	H21B—C21—H21C	109.5
O3—C12—O4	127.40 (17)	C19—C22—H22A	109.5
O3—C12—N3	123.38 (15)	C19—C22—H22B	109.5
O4—C12—N3	109.21 (14)	H22A—C22—H22B	109.5
O2—C13—C14	109.60 (13)	C19—C22—H22C	109.5
O2—C13—C16	101.24 (15)	H22A—C22—H22C	109.5
C14—C13—C16	111.47 (16)	H22B—C22—H22C	109.5
O2—C13—C15	109.64 (13)		
			
C13—O2—C1—O1	−2.0 (2)	C12—N3—C8—C7	2.4 (2)
C13—O2—C1—N1	176.90 (12)	C11—N3—C8—C7	179.74 (15)
C2—N1—C1—O1	179.68 (15)	N2—C7—C8—C9	−0.3 (2)
C5—N1—C1—O1	0.9 (2)	N2—C7—C8—N3	179.67 (14)
C2—N1—C1—O2	0.7 (2)	N3—C8—C9—C10	0.08 (18)
C5—N1—C1—O2	−178.04 (13)	C7—C8—C9—C10	−179.92 (16)
C1—N1—C2—C3	−178.48 (14)	C8—C9—C10—C11	0.1 (2)
C5—N1—C2—C3	0.47 (17)	C9—C10—C11—N3	−0.29 (19)
N1—C2—C3—C4	−0.43 (17)	C12—N3—C11—C10	177.71 (15)
C2—C3—C4—C5	0.25 (18)	C8—N3—C11—C10	0.34 (19)
C3—C4—C5—N1	0.04 (17)	C19—O4—C12—O3	13.9 (2)
C3—C4—C5—C6	178.28 (15)	C19—O4—C12—N3	−166.59 (12)
C2—N1—C5—C4	−0.31 (16)	C11—N3—C12—O3	−178.45 (15)
C1—N1—C5—C4	178.64 (14)	C8—N3—C12—O3	−1.5 (2)
C2—N1—C5—C6	−178.67 (14)	C11—N3—C12—O4	2.0 (2)
C1—N1—C5—C6	0.3 (2)	C8—N3—C12—O4	178.95 (13)
C7—N2—C6—C5	−147.26 (14)	C1—O2—C13—C14	65.63 (19)
C17—N2—C6—C5	87.35 (17)	C1—O2—C13—C16	−176.54 (14)
C4—C5—C6—N2	6.7 (2)	C1—O2—C13—C15	−59.2 (2)
N1—C5—C6—N2	−175.38 (13)	C7—N2—C17—C18	71.9 (2)
C6—N2—C7—C8	80.67 (16)	C6—N2—C17—C18	−163.52 (15)
C17—N2—C7—C8	−155.20 (14)	C12—O4—C19—C22	177.50 (13)
C12—N3—C8—C9	−177.58 (15)	C12—O4—C19—C21	−64.16 (18)
C11—N3—C8—C9	−0.26 (18)	C12—O4—C19—C20	60.31 (18)

### Hydrogen-bond geometry (Å, º)

*Cg*2 is the centroid of the N3/C8–C11 pyrrole ring.

**Table:**

*D*—H···*A*	*D*—H	H···*A*	*D*···*A*	*D*—H···*A*
C14—H14*A*···O1	0.98	2.48	3.062 (3)	118
C15—H15*C*···O1	0.98	2.45	2.989 (2)	114
C20—H20*A*···O3	0.98	2.54	3.100 (3)	116
C21—H21*C*···O3	0.98	2.43	3.012 (3)	118
C16—H16*B*···*Cg*2^i^	0.98	2.85	3.779 (2)	158

Symmetry code: (i) *x*, *y*, *z*−1.
